# The association of hemoglobin levels and balance function in patients with stroke: a multicenter study in China

**DOI:** 10.3389/fnins.2025.1759185

**Published:** 2026-01-07

**Authors:** Jie Zhu, Ranran Bi, Shuyang Zhang, Yansheng Lin, Yifang Lin, Xuezhen Zhao, Jiali Lin, Jie Jia

**Affiliations:** 1 Department of Rehabilitation Medicine, Huashan Hospital, Fudan University, Shanghai, China; 2 National Clinical Research Center for Aging and Medicine, Huashan Hospital, Fudan University, Shanghai, China; 3 Department of Rehabilitation Medicine, Shanghai East Hospital, School of Medicine, Tongji University, Shanghai, China; 4 Department of Rehabilitation Medicine, The First Affiliated Hospital of Fujian Medical University, Fuzhou, China; 5 Department of Rehabilitation Medicine, Fujian University of Traditional Chinese Medicine, Fuzhou, China

**Keywords:** balance impairment, Berg balance scale, hemoglobin, multicenter, stroke

## Abstract

**Background:**

Balance impairment following stroke is a leading cause of disability and falls. Hemoglobin (Hb) affects systemic and cerebral oxygen delivery and may influence neuromuscular function and post-stroke balance, but evidence from large multicenter clinical samples is limited. We investigated the association between hemoglobin concentration and balance performance in a Chinese multicenter cross-sectional study of stroke patients.

**Methods:**

We studied 2,006 neuroimaging-confirmed stroke patients from 26 hospitals. Balance impairment was defined as BBS ≤ 40. Admission Hb (g/dL) was analyzed per 1 g/dL and by tertiles (<12.6 g/dL, 12.6–14.0 g/dL, ≥14.0 g/dL). Multivariable logistic regression with sequential adjustment, restricted cubic splines, and prespecified subgroup and sensitivity analyses evaluated associations.

**Results:**

Balance impairment occurred in 70.5% (1,414/2,006). Each 1 g/dL higher Hb was associated with lower odds of impairment in unadjusted (OR 0.83, 95% CI 0.78–0.87; *p* < 0.001) and fully adjusted models (OR 0.89, 95% CI 0.83–0.96; *p* = 0.002). This association remained robust after comprehensive adjustment for demographic, lifestyle, comorbidity, stroke characteristics, and lesion location factors. Compared with the lowest tertile, adjusted ORs were 0.72 (95% CI 0.53–0.99; *p* = 0.042) for the middle tertile and 0.62 (95% CI 0.45–0.85; *p* = 0.003) for the highest tertile. Spline analyses suggested a broadly linear inverse association; results were consistent across subgroups and sensitivity checks.

**Conclusion:**

Higher admission hemoglobin was independently associated with better balance after stroke. Prospective studies should test whether Hb optimization improves rehabilitation outcomes.

## 1 Introduction

Stroke is a leading cause of death and long-term disability worldwide, with particularly high incidence and burden in China where aging and vascular risk factors have driven rising prevalence and substantial socioeconomic impact (GBD 2023 Causes of Death Collaborators, 2025; Zhao et al., 2023; Ma et al., 2021). Impaired postural control and balance are central determinants of mobility, fall risk, and independence after stroke, and they strongly predict community reintegration and quality of life (Smith et al., 2017; Wade and Hewer, 1987). Identifying modifiable systemic contributors to balance impairment is therefore a priority for rehabilitation (Allison et al., 2011).

Hemoglobin concentration is a key determinant of systemic oxygen delivery and muscle perfusion; both low and excessively high hemoglobin have been associated with adverse neurological outcomes in cerebrovascular disease (Barlas et al., 2016; Zhang et al., 2021; Kellert et al., 2011). In older adults and chronic disease populations, anemia correlates with reduced muscle strength, slower gait speed, and poorer balance performance (Aung et al., 2011; Wang and Lin, 2024; Corona et al., 2022). Emerging stroke-specific evidence links lower hemoglobin or anemia on admission to larger infarct volumes, worse early neurological deficits, higher mortality, and poorer functional recovery (Lin et al., 2023). However, most studies focus on global functional scales (mRS, NIHSS) or mortality rather than quantitative measures of balance; investigations of hemoglobin and specific domains of motor recovery are sparse and frequently limited by single-center design, small samples, or cross-sectional analyses (Altersberger et al., 2020; He et al., 2020; Lasek-Bal et al., 2015).

The relationship between hemoglobin levels and balance function after stroke remains poorly characterized in large, diverse populations, and the potential for hemoglobin optimization to improve rehabilitation outcomes is unexplored. To address this, we conducted a multicenter study in China to examine the association between hemoglobin concentration and objective balance measures in patients with stroke, adjusting for infarct characteristics, comorbidity burden, and rehabilitation intensity. This approach aims to clarify a potentially modifiable systemic factor that may inform individualized rehabilitation strategies and secondary prevention.

## 2 Materials and methods

### 2.1 Study design and ethical considerations

This cross-sectional, multicenter study enrolled patients with neuroimaging-confirmed stroke who underwent standardized rehabilitation assessments at tertiary hospitals across China. The protocol was approved by the Ethics Review Committee of Huashan Hospital, Fudan University (approval number: HIRB2022-510) and the study was registered in the Chinese Clinical Trial Registry (registration number: ChiCTR2200063611). The study was conducted in accordance with the Declaration of Helsinki, reported following STROBE guidelines (von Elm et al., 2007), and requirements for informed consent were determined, obtained, or waived by the respective local IRBs as appropriate; all patient data were de-identified prior to analysis.

### 2.2 Participants and data sources

**Figure 1:**
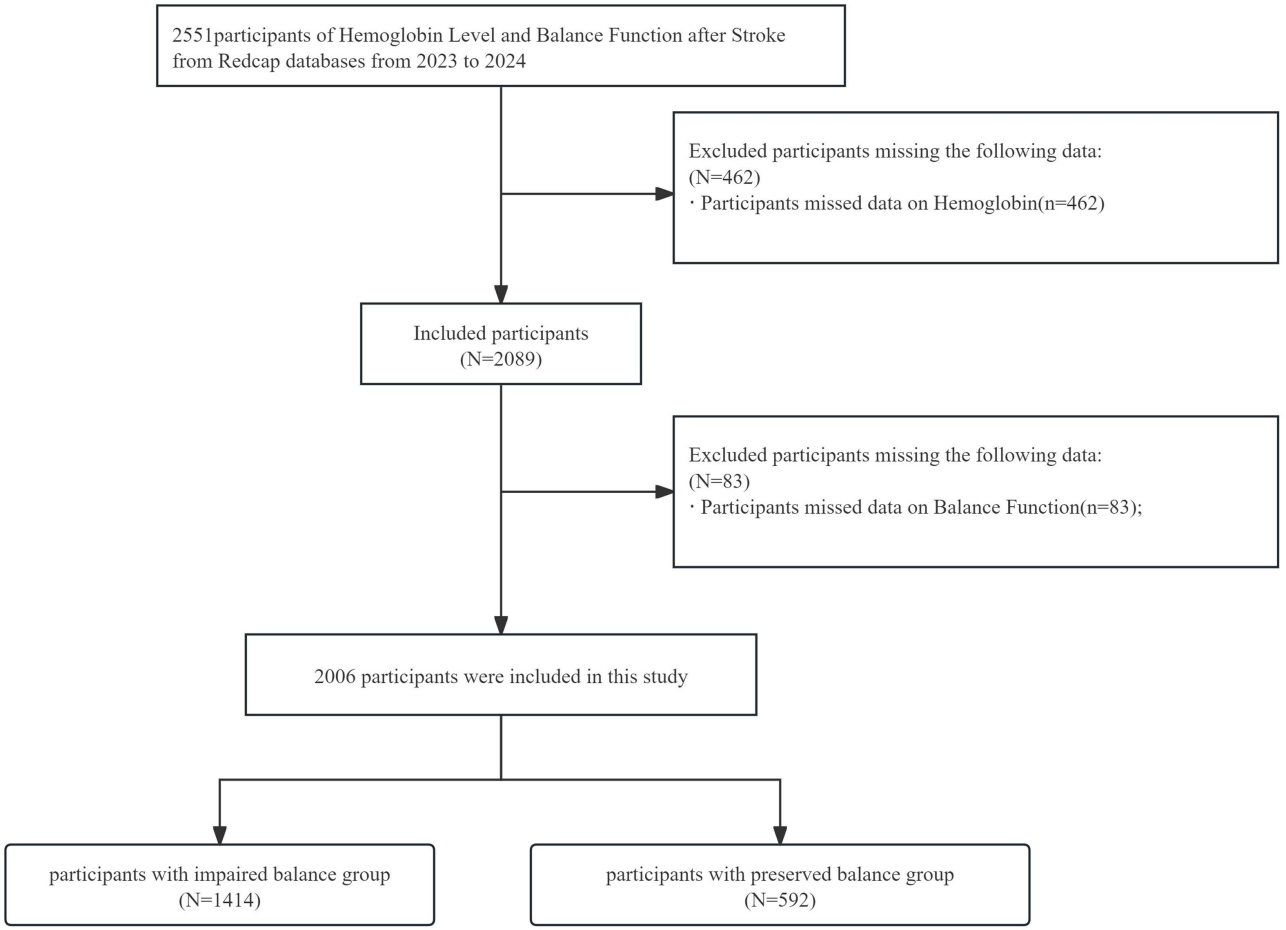
Flowchart of participant inclusion.

Consecutive adult patients (age ≥18 years) admitted with a primary diagnosis of ischemic or hemorrhagic stroke and referred for in-hospital rehabilitation evaluation between January 2023 and December 2024 were eligible (Figure 1). Demographic data, stroke characteristics, vascular risk factors, comorbidities and laboratory results were abstracted from electronic medical records by trained research staff at each site using a centralized case report form. Clinical and rehabilitation assessments—including standardized functional scales (e.g., Berg Balance Scale, Fugl-Meyer Assessment, Brunnstrom stage) and other performance measures—were administered following site-specific rater training. Data entry personnel received multiple rounds of centralized training to ensure consistency; all data were entered into a REDCAP^1^ database with built-in range and consistency checks and managed centrally with routine monitoring and query resolution prior to analysis.

### 2.3 Inclusion and exclusion criteria

Inclusion criteria were: (1) neuroimaging-confirmed stroke; (2) hemoglobin measurement obtained on admission and completion of rehabilitation assessments within 7 days of the laboratory draw; and (3) ability to participate in standardized balance testing (with or without assistance).

Exclusion criteria were any clinical conditions that, in the treating clinician’s judgment, could substantially confound assessment or precipitate clinical deterioration during evaluation, including: (1) severe or poorly controlled hypertension, advanced heart failure, severe active infection (e.g., pneumonia), diabetic ketoacidosis, uncontrolled or frequent seizures, or other acute medical problems likely to worsen during assessment; and (2) uncontrolled systemic disorders such as overt thyroid dysfunction (hyper- or hypothyroidism), severe hepatic or renal impairment (including dialysis dependence), active acute rheumatologic disease, clinically significant electrolyte imbalances. Records with missing key covariates or outcomes, implausible values, or duplicate entries were excluded after data cleaning, yielding the final analytic cohort.

### 2.4 Hemoglobin measurement

Peripheral venous blood was collected on hospital admission as part of standard care and analyzed in each site’s clinical laboratory using automated hematology analyzers. Hemoglobin (Hb) concentration was recorded in g/dL. Hemoglobin concentration was measured in g/dL (For reference, 1 g/dL = 10 g/L). For primary analyses Hb was modeled both as a continuous variable and study-specific tertiles.

### 2.5 Balance and motor function assessments

Balance was assessed with the Berg Balance Scale (BBS), a validated 14-item performance measure with scores ranging 0–56; higher scores indicate better balance (Ma et al., 2021). Based on established cutoffs, total BBS > 40 was classified as preserved balance and ≤40 as balance impairment with increased fall risk (Berg et al., 1995; Alghadir et al., 2018). Lower limb motor impairment was evaluated using the Fugl-Meyer Assessment for the lower extremity (FMA-LE), scored 0–34, a standardized measure of motor control and movement synergy patterns (Gladstone et al., 2002), which was dichotomized at the median score (Pandian et al., 2016) for comparative analyses. Motor recovery stage was categorized using Brunnstrom staging (stages 1–6; Brunnstrom, 1966). All raters completed standardized training and inter-rater reliability checks were performed in a subset of subjects.

### 2.6 Covariates

Potential confounders were prespecified on the basis of biological plausibility, clinical relevance and prior literature. The following covariates were entered into sequential regression models: demographic factors—age (continuous), sex (male/female) and education level (categorical: below high school / high school / college or above); lifestyle factors—smoking status (yes/no) and alcohol use (yes/no); anthropometry—BMI (continuous); vascular comorbidities—hypertension (yes/no), diabetes mellitus(DM; yes/no), Coronary heart disease(CAD; yes/no) and prior stroke (yes/no); stroke characteristics—type (ischemic/hemorrhagic) and days from stroke onset to assessment (Day_of_illness, continuous); lesion topography—presence of basal ganglia(BG), cerebellum(CB) or brainstem(BS) lesions on neuroimaging (binary, recorded by neuroradiologists); and motor function—Brunnström lower-limb stage (ordinal, I–VI) and FMA-LE (continuous).

### 2.7 Statistical analysis

**Figure 2:**
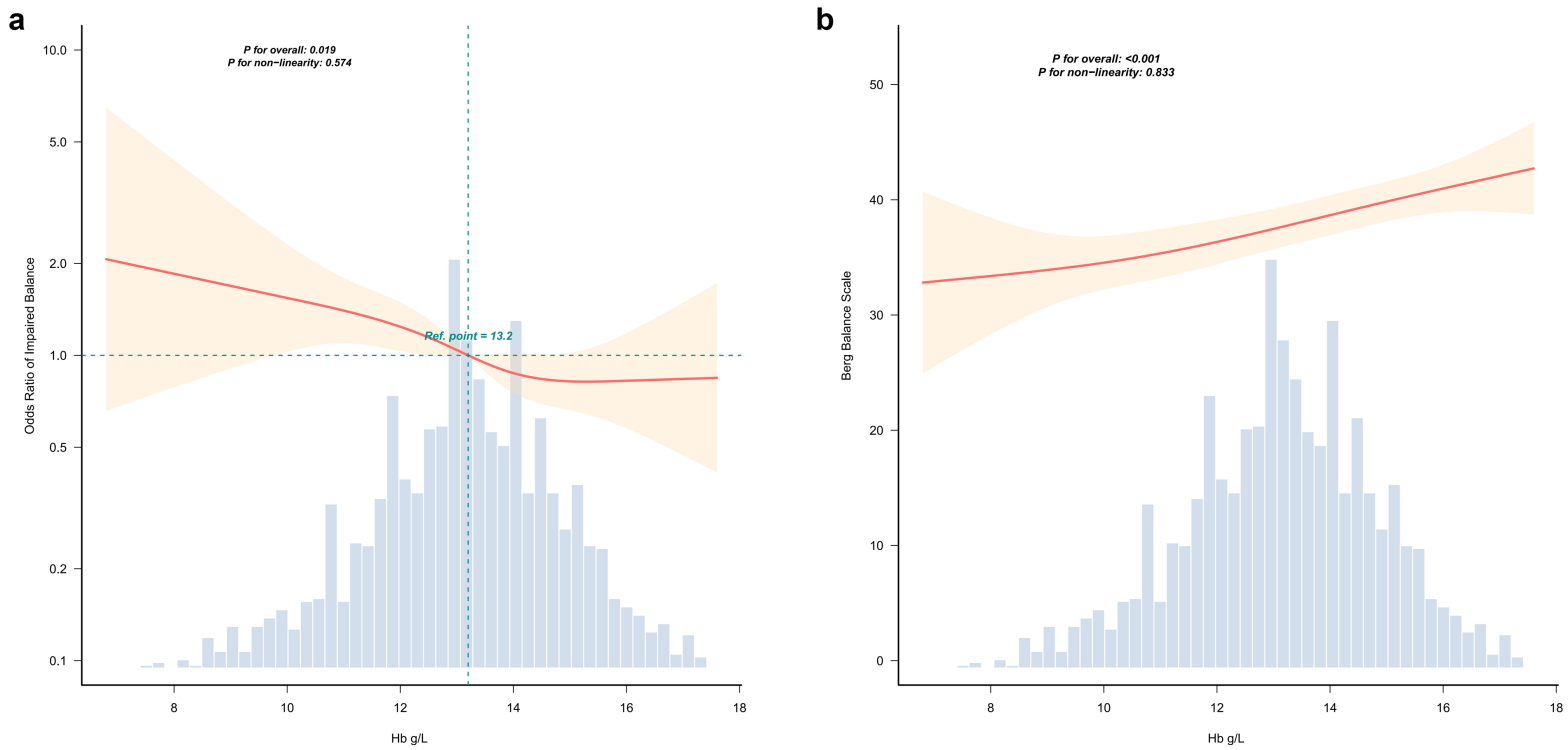
Restricted cubic spline analyze between Hb and balance function. Dose–response relationship assessed via linear regression after confirming linearity with restricted cubic splines, adjusted for all covariates specified in model 4. **(a)** Spline for the dichotomous outcome of balance impairment, using Hb = 13.2 g/dL as the reference point (*P* for non-linearity = 0.574). **(b)** Spline for the continuous balance measure (*P* for non-linearity = 0.833). The *p* value for the overall association was < 0.05.

**Figure 3:**
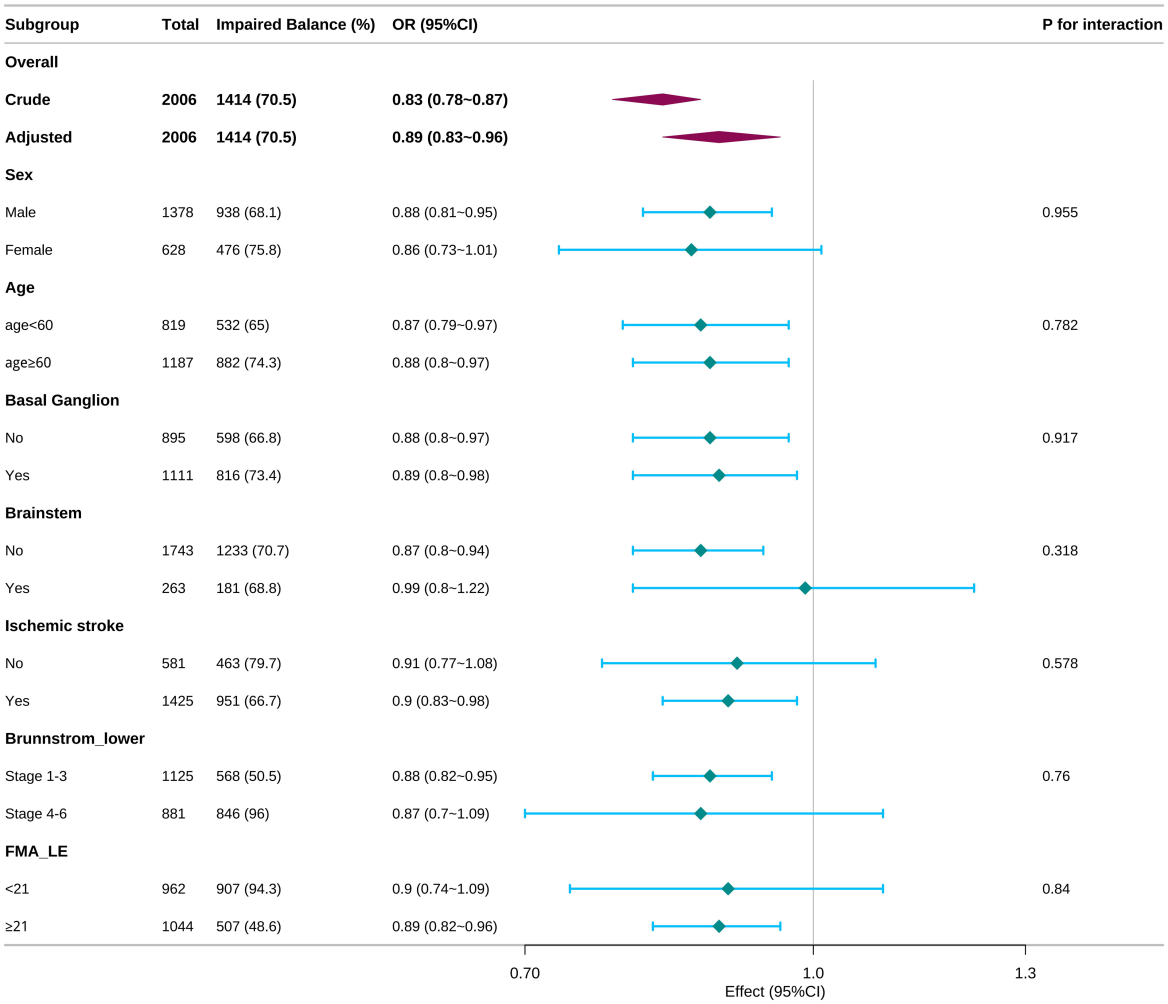
Stratified multivariable analysis of the association between Hb and impaired balance according to baseline characteristics. Subgroup interaction analyses (sex, age, lesion locations, stroke subtype, severity of lower limb functional impairment). Each stratification adjusts for all factors (age, sex, BG, BS, ischemic stroke, Brunnstrom, FMA-LE) except for the stratification factor itself.

Statistical analyses were performed to examine the association between admission hemoglobin (Hb) and balance (BBS as both a continuous outcome and dichotomized at ≤40). Variable distributions were inspected with histograms, Q–Q plots and the Kolmogorov–Smirnov test; normally distributed continuous variables are reported as mean ± SD and compared by independent samples t-tests (e.g., age, Hb), skewed variables as median (IQR) and compared by the Mann–Whitney U test (e.g., BBS), and categorical variables as n (%) with comparisons by χ2 or Fisher’s exact test as appropriate (Table 1). Primary models comprised multivariable linear regression for continuous BBS and logistic regression for dichotomous balance impairment, with Hb entered both as a continuous term (per 1 g/dL and per SD) and categorically by quantiles. Four prespecified adjustment sequences were fitted: Model 1 unadjusted; Model 2 adjusted for age, sex and education; Model 3 additionally adjusted for smoking, alcohol use, BMI, hypertension, DM, CAD, prior stroke and Day of illness; and Model 4 further adjusted for time from stroke type, BG, BS and CB lesion indicators, Brunnstrom lower-limb stage and FMA-LE (Table 2). The dose–response relationship was evaluated using linear regression, following confirmation of linearity by a restricted cubic spline model (Figure 2). Interaction analyses were conducted in prespecified subgroups (sex, age, lesion locations, stroke subtype, and severity of lower limb functional impairment; Figure 3). Missing covariate data were handled by multiple imputation using chained equations (m = 5) via the R mice package, and all analyses were repeated in the complete-case cohort for comparison. Effect estimates and *p*-values from all models were reported. All tests were two-sided with *α* = 0.05, and analyses were conducted in R (version 4.2.3) and the Free Statistics platform.

**Table 1:** Baseline characteristics according to balance function in stroke patients.

Variables	Total (*n* = 2006)	Impaired balance (*n* = 1,414)	Preserved balance (*n* = 592)	*p*
Sex, n (%)				< 0.001
Male	1,378 (68.7)	938 (66.3)	440 (74.3)	
Female	628 (31.3)	476 (33.7)	152 (25.7)	
Age, years	61.8 ± 12.4	62.9 ± 12.2	59.3 ± 12.5	< 0.001
BMI, kg/m^2^	23.9 ± 3.1	23.9 ± 3.1	24.0 ± 2.9	0.339
Education, n (%)				< 0.001
Below high school	1,356 (67.6)	998 (70.6)	358 (60.5)	
High school graduate	373 (18.6)	240 (17)	133 (22.5)	
College or above	277 (13.8)	176 (12.4)	101 (17.1)	
Smoke, n (%)				0.902
No	606 (30.2)	426 (30.1)	180 (30.4)	
Yes	1,400 (69.8)	988 (69.9)	412 (69.6)	
Drink, n (%)				0.644
No	351 (17.5)	251 (17.8)	100 (16.9)	
Yes	1,655 (82.5)	1,163 (82.2)	492 (83.1)	
Hypertension, n (%)				0.269
No	729 (36.3)	503 (35.6)	226 (38.2)	
Yes	1,277 (63.7)	911 (64.4)	366 (61.8)	
CAD, n (%)				0.507
No	1908 (95.1)	1,342 (94.9)	566 (95.6)	
Yes	98 (4.9)	72 (5.1)	26 (4.4)	
Previous stroke, n (%)				0.238
No	1863 (92.9)	1,307 (92.4)	556 (93.9)	
Yes	143 (7.1)	107 (7.6)	36 (6.1)	
DM, n (%)				0.006
No	1809 (90.2)	1,292 (91.4)	517 (87.3)	
Yes	197 (9.8)	122 (8.6)	75 (12.7)	
Day_of_illness	30.0 (15.0, 68.0)	30.0 (15.0, 63.8)	32.0 (14.0, 82.0)	0.731
BG, n (%)				0.001
No	895 (44.6)	598 (42.3)	297 (50.2)	
Yes	1,111 (55.4)	816 (57.7)	295 (49.8)	
BS, n (%)				0.232
No	1921 (95.8)	1,359 (96.1)	562 (94.9)	
Yes	85 (4.2)	55 (3.9)	30 (5.1)	
CB, n (%)				0.525
No	1743 (86.9)	1,233 (87.2)	510 (86.1)	
Yes	263 (13.1)	181 (12.8)	82 (13.9)	
Ischemic stroke, n (%)				< 0.001
No	581 (29.0)	463 (32.7)	118 (19.9)	
Yes	1,425 (71.0)	951 (67.3)	474 (80.1)	
Hb(g/dl)	13.2 ± 1.8	13.0 ± 1.8	13.6 ± 1.7	< 0.001
Brunnstrom_lower, n (%)				< 0.001
Stage 1–3	1,125 (56.1)	568 (40.2)	557 (94.1)	
Stage 4–6	881 (43.9)	846 (59.8)	35 (5.9)	
FMA_LE, Score (0–66)				< 0.001
<21	962 (48.0)	907 (64.1)	55 (9.3)	
≥21	1,044 (52.0)	507 (35.9)	537 (90.7)	
BBS, Score (0–56)	21.0 (4.0, 42.0)	8.0 (2.0, 23.0)	49.0 (44.0, 55.0)	< 0.001

CAD, Coronary heart disease; DM, Diabetes mellitus; BG, Basal ganglia; BS, Brainstem; CB, Cerebellum; Hb, Hemoglobin; FMA_LE, Fugl-Meyer Assessment of Lower Extremity Motor Function; BBS, Berg Balance Scale. Data are presented as mean ± standard deviation (SD) for normally distributed continuous variables (independent samples t-test), median with interquartile range (IQR) for skewed variables (Mann–Whitney U test), and number with percentage for categorical variables (χ^2^ or Fisher’s exact test).

**Table 2:** Multiple logistic regression analysis of factors associated with HB and BBS.

Variable	N total	N event (%)	Model 1		Model 2		Model 3		Model 4	
			OR (95%CI)	*p* value	OR (95%CI)	*p* value	OR (95%CI)	*p* value	OR (95%CI)	*p* value
Hb(g/dl)	2006	1,414 (70.5)	0.83 (0.78–0.87)	<0.001	0.85 (0.81–0.91)	<0.001	0.84 (0.79–0.9)	<0.001	0.89 (0.83–0.96)	0.002
Hb(<12.6 g/dl)	481	375 (78)	1(Ref)		1(Ref)		1(Ref)		1(Ref)	
Hb(12.6-14 g/dl)	533	367 (68.9)	0.63 (0.49–0.82)	<0.001	0.65 (0.51–0.84)	0.001	0.63 (0.49–0.82)	<0.001	0.72 (0.53–0.99)	0.042
Hb(≥14 g/dL)	513	307 (59.8)	0.42 (0.33–0.54)	<0.001	0.48 (0.37–0.62)	<0.001	0.46 (0.35–0.6)	<0.001	0.62 (0.45–0.85)	0.003
*P* for trend				<0.001		<0.001		<0.001		0.003

OR, Odds ratio; CI, confidence interval; Ref, reference. Primary models: Linear regression for continuous BBS and logistic regression for dichotomous balance impairment. Hb was parameterized as continuous (per 1 g/dL and per SD) and categorical (tertiles). Four prespecified adjustment levels were implemented hierarchically: Model 1 (unadjusted), Model 2 (adjusted for sex, age, education), Model 3 (adjusted for all covariates in model 2 plus smoke, drink, BMI, hypertension, DM, CAD, Previous stroke, Day_of_illness), Model 4 (adjusted for all covariates in model 3 plus stroke type, BG, basal ganglia; BS, brainstem; CB, cerebellum; Brunnstrom_lower, FMA_LE).

## 3 Results

Table 1 summarizes baseline characteristics of the 2,006 stroke patients stratified by balance status (impaired, *n* = 1,414; preserved, *n* = 592). Patients with impaired balance were older (62.9 ± 12.2 vs. 59.3 ± 12.5 years; *p* < 0.001) and included a higher proportion of women (33.7% vs. 25.7%; *p* < 0.001). Educational attainment differed between groups, with more patients below high school level in the impaired group (70.5% vs. 60.1%; *p* < 0.001). There were no significant between-group differences in BMI, smoking, alcohol use, hypertension, CAD, prior stroke, or the CB and BS categories (all *p* > 0.05). DM was more frequent among patients with preserved balance (12.7% vs. 8.6%; *p* = 0.006). BG was more common in the impaired group (57.7% vs. 49.8%; *p* = 0.001), and ischemic stroke was more prevalent in the preserved group (80.1% vs. 67.3%; *p* < 0.001). Mean admission hemoglobin concentration differed significantly between groups (13.0 ± 1.8 g/dL in impaired vs. 13.6 ± 1.7 g/dL in preserved; *p* < 0.001). Patients with impaired balance had markedly worse motor and functional profiles: lower Brunnstrom lower-limb stages (*p* < 0.001), lower FMA-LE scores (all *p* < 0.001), substantially lower BBS (median 8.0 vs. 49.0; *p* < 0.001).

Table 2 presents the association between admission Hb and balance impairment (BBS ≤ 40) in 2,006 patients (1,414 events, 70.5%). In unadjusted analysis each 1 g/dL increase in Hb was associated with a 17% lower odds of balance impairment (OR 0.83, 95% CI 0.78–0.87; *p* < 0.001); this association remained essentially unchanged after sequential adjustment for demographic factors, stroke characteristics, comorbidities and motor impairment (fully adjusted OR 0.89, 95% CI 0.83–0.96; *p* = 0.002), corresponding to a 11% risk reduction per 1 g/dL. When Hb was modeled categorically, patients in the mid tertile (12.6–14.0 g/dL) had 37% lower odds of balance impairment compared with the lowest tertile (<12.6 g/dL) in both unadjusted (OR 0.63, 95% CI 0.49–0.82; *p* < 0.001) and adjusted models (OR 0.72, 95% CI 0.53–0.99; *p* = 0.042). Those in the highest tertile (≥14 g/dL) had the greatest risk reduction (unadjusted OR 0.42, 95% CI 0.33–0.54, *p* < 0.001; adjusted OR 0.62, 95% CI 0.45–0.85, *p* = 0.003), corresponding to 38–58% lower odds versus the lowest tertile. A significant dose–response relationship was evident (p for trend < 0.05), and findings were robust across all adjustment models.

Restricted cubic spline analyses (*n* = 2,006) evaluated the dose–response relation between admission hemoglobin (Hb, g/dL) and balance outcomes; plots were truncated at the 99.5th percentile of Hb to minimize influence of extreme values. Figure 2A displays the adjusted spline for the dichotomous balance outcome (balance impairment vs. preserved), using Hb = 13.2 g/dL as the referent (P for non-linearity = 0.574); Figure 2B shows the corresponding spline for the continuous balance measure. The overall association was highly significant (P for overall < 0.05) whereas the test for nonlinearity was not (P for non-linearity = 0.833), indicating that the relationship is consistent with a linear dose–response across the observed Hb range.

Figure 3 presents stratified (subgroup) analyses of the adjusted association between admission hemoglobin and balance impairment. The inverse relationship observed in the overall cohort (adjusted OR 0.89, 95% CI 0.83–0.96; P for overall <0.001) was generally consistent across clinically relevant strata. Point estimates favored an inverse association of higher hemoglobin in both sexes (male OR 0.88, female OR 0.86) and in younger (<60 years OR 0.87) and older (≥60 years OR 0.88) patients. Similar effect sizes were observed regardless of Brunnstrom strata and FMA-LE. Lesion location (basal ganglia and brainstem) did not materially modify the association; the cerebellar lesion subgroup also showed no significant effect modification. Although confidence intervals were wider in smaller subgroups, no subgroup showed a statistically significant interaction (all P for interaction >0.05).

### 3.1 Sensitivity analyses

To evaluate the robustness of our primary finding—that lower hemoglobin levels are independently associated with poorer balance function—we conducted several sensitivity analyses. When we excluded patients with missing covariates (*n* = 1,527; events = 1,049, 68.7%), the association between hemoglobin levels and balance function remained statistically significant and directionally consistent (see Supplementary Table S1 for detailed results). These results indicate that our main finding is not overly dependent on a specific analytical choice or patient subset.

## 4 Discussion

In this multicenter cross-sectional study of 2,006 patients with stroke undergoing standardized rehabilitation evaluation, we observed a robust inverse association between admission hemoglobin (Hb) concentration and the presence of balance impairment. Higher Hb was associated with lower odds of balance dysfunction across analytic approaches: in multivariable models each 1 g/dL increment in Hb was associated with a clinically meaningful reduction in the odds of balance impairment, and patients in the mid and highest Hb tertiles had substantially lower adjusted odds compared with those in the lowest tertile. Restricted cubic spline analyses indicated a broadly linear relationship across the observed Hb range, and the association persisted in sensitivity analyses excluding patients with missing covariates. Pre-specified subgroup analyses did not identify statistically significant interactions by age, sex, prior stroke or lesion location (BG, BS and CB), suggesting the inverse Hb-balance association was consistent across major clinical strata. Given the relatively low number of patients with isolated cerebellar infarction in our cohort, which may limit statistical power in this subgroup, further large-scale studies specifically targeting stroke patients with isolated cerebellar involvement are warranted to confirm these observed trends and fully elucidate the role of Hb in their specific balance recovery pathway.

Our findings extend previous work linking anemia or low Hb with worse post-stroke outcomes. Prior cohort and registry studies have reported that lower Hb on admission is associated with larger infarct size, greater early neurological deficit and worse global functional outcome or mortality after ischemic stroke (Khan et al., 2018; Hao et al., 2013; Ceulemans et al., 2024). In non-stroke older populations, anemia has been associated with reduced muscle strength, slower gait speed and increased fall risk—domains that overlap substantially with balance impairment assessed in rehabilitation settings (Cecchi et al., 2017; Joosten et al., 2016; Girelli et al., 2018). Several single-center stroke studies have suggested that lower Hb predicts poorer rehabilitation gains, but most prior investigations focused on global outcome scales (e.g., mRS, discharge destination) rather than standardized balance performance (Berg Balance Scale; He et al., 2020; Li et al., 2016; Naess et al., 2019). By using a large, multicenter sample with standardized balance and motor assessments, our study provides direct evidence linking Hb concentration to a rehabilitation-relevant impairment—postural control—that is tightly coupled to mobility, fall risk and independence.

If the Hb-balance relation is causal or partly causal, Hb optimization could represent a modifiable target to enhance post-stroke balance and reduce fall risk. Routine monitoring of Hb as part of comprehensive rehabilitation assessment is readily implementable and may help identify patients at higher risk of balance impairment who could benefit from targeted nutritional, hematologic or exercise-based interventions. However, evidence from interventional studies is limited. Transfusion trials and erythropoiesis-stimulating agent studies in stroke have focused primarily on mortality and neurological endpoints with mixed results and safety concerns; none have specifically evaluated balance or rehabilitation outcomes. Thus, our findings support the rationale for prospective studies and pilot intervention trials testing whether correction of clinically significant anemia—or broader strategies to improve oxygen delivery and muscle function—can translate into measurable gains in balance, mobility and participation.

Longitudinal cohort studies with repeated Hb and comprehensive physiologic assessment (including measures of muscle oxygenation, iron status, inflammatory biomarkers and objective mobility monitoring) would clarify temporality and mechanisms. Randomized pilot trials targeting iron deficiency or anemia correction in selected post-stroke populations, with balance and fall risk as predefined rehabilitation endpoints, are needed to evaluate efficacy and safety. Integration of imaging biomarkers and neuromuscular phenotyping could identify subgroups most likely to benefit from systemic versus targeted neurorehabilitation strategies.

This study has important limitations. Its cross-sectional design precludes causal inference and cannot rule out reverse causation. Residual confounding by unmeasured factors (nutrition, inflammatory markers, iron status, transfusion history, and chronic disease severity) is possible despite multivariable adjustment and sensitivity analyses. Hemoglobin was measured once on admission, preventing assessment of temporal trajectories or differentiation of acute versus chronic anemia. Evaluations were performed at variable post-stroke intervals across multiple centers; although we adjusted for time from onset and accounted for clustering, heterogeneity in care and rehabilitation intensity may have affected outcomes. Uniform imaging metrics such as infarct volume and network disruption were unavailable, limiting mechanistic interpretation. Furthermore, while we adjusted for major motor impairment, potential residual confounding from unmeasured or more nuanced factors influencing balance, such as subtle deficits in sensorimotor integration or proprioception, cannot be excluded.

## 5 Conclusion

In this large multicenter sample of patients undergoing stroke rehabilitation evaluations, higher admission hemoglobin was inversely associated with lower odds of balance impairment, with a broadly linear dose–response and consistent findings across sensitivity and subgroup analyses. These results highlight hemoglobin as a potentially modifiable systemic correlate of post-stroke balance dysfunction and motivate prospective studies to determine whether Hb optimization can improve rehabilitation outcomes and reduce fall risk.

## Data Availability

The original contributions presented in the study are included in the article/Supplementary material, further inquiries can be directed to the corresponding author.

## Footnotes

1
https://himedc.huashan.org.cn:5288/
