# Supplementary material for: The association of hemoglobin levels and balance function in patients with stroke: a multicenter study in China
Source: Front Neurosci. 2026 Jan 7;19:1759185. doi: 10.3389/fnins.2025.1759185 (PMC12819781; doi:10.3389/fnins.2025.1759185)
Supplement: Supplementary file 1 [file Table_1.docx]

**Table S1. Multiple logistic regression analysis of factors associated with HB and BBS.**

| **Variable** | **N total** | **N event (%)** | **Model 1** | | **Model 2** | | **Model 3** | | **Model 4** | |
| --- | --- | --- | --- | --- | --- | --- | --- | --- | --- | --- |
|  |  |  | **OR (95%CI)** | ***P value*** | **OR (95%CI)** | ***P value*** | **OR (95%CI)** | ***P value*** | **OR (95%CI)** | ***P value*** |
| Hb(g/dL) | 1527 | 1049 (68.7) | 0.84 (0.78~0.89) | <0.001 | 0.87 (0.81~0.93) | <0.001 | 0.86 (0.8~0.92) | <0.001 | 0.91 (0.83~0.99) | 0.022 |
| Lbhb(<12.6g/dL) | 481 | 375 (78) | 1(Ref) |  | 1(Ref) |  | 1(Ref) |  | 1(Ref) |  |
| Lbhb(12.6-14.1g/dL) | 533 | 367 (68.9) | 0.62 (0.47~0.83) | 0.001 | 0.64 (0.48~0.85) | 0.002 | 0.62 (0.46~0.83) | 0.001 | 0.73 (0.51~1.03) | 0.076 |
| Lbhb(≥14.1g/dL) | 513 | 307 (59.8) | 0.42 (0.32~0.56) | <0.001 | 0.48 (0.36~0.65) | <0.001 | 0.46 (0.34~0.62) | <0.001 | 0.60 (0.42~0.87) | 0.007 |
| *P* for trend |  |  |  | <0.001 |  | <0.001 |  | <0.001 |  | <0.001 |

**Model 1:** unadjusted.

**Model 2:** adjusted for sex, age, education.

**Model 3:** adjusted for all covariates in model 2 plus smoke, drink, BMI, hypertension, DM, CAD, Previous stroke, Day_of_illness.

**Model 4:** adjusted for all covariates in model 3 plus stroke type, BG, BS, CB, Brunnstrom_lower, FMA_LE.

**Abbreviations:** OR, Odds ratio; CI, confidence interval; Ref, reference.

**Supplementary Table S1** reports the sensitivity analysis conducted after exclusion of patients with missing covariates (n = 1,527; events = 1,049, 68.7%). In the analysis treating hemoglobin (Hb, g/dL) as a continuous variable, each 1 g/dL increase in Hb was associated with an unadjusted OR of 0.84 (95% CI 0.78–0.89; p < 0.001) (Model 1). Sequential adjustment yielded similar estimates: Model 2 OR 0.87 (95% CI 0.81–0.93; p < 0.001); Model 3 OR 0.86 (95% CI 0.8–0.92; p < 0.001); and Model 4 OR 0.91 (95% CI 0.83–0.99; p = 0.022). When Hb was categorized by tertiles using the lowest group (<12.6 g/dL) as reference, the middle tertile (12.6–14.1 g/dL) had ORs of 0.62 (95% CI 0.47–0.83; p = 0.001) in Model 1, 0.64 (95% CI 0.48–0.85; p = 0.002) in Model 2, 0.62 (95% CI 0.46–0.83; p = 0.001) in Model 3, and 0.73 (95% CI 0.51–1.03; p = 0.076) in Model 4. The highest tertile (≥14.1 g/dL) had ORs of 0.42 (95% CI 0.32–0.56; p < 0.001) in Model 1, 0.48 (95% CI 0.36–0.65; p < 0.001) in Model 2, 0.46 (95% CI 0.34–0.62; p < 0.001) in Model 3, and 0.60 (95% CI 0.42–0.87; p < 0.001) in Model 4. A significant monotonic trend across Hb tertiles was observed in all models (P for trend < 0.001)
